# Intentional Rounding versus Standard of Care for Patients Hospitalised in Internal Medicine Wards: Results from a Cluster-Randomised Nation-Based Study

**DOI:** 10.3390/jcm11143976

**Published:** 2022-07-08

**Authors:** Dino Stefano Di Massimo, Gianluca Catania, Annachiara Crespi, Andrea Fontanella, Dario Manfellotto, Micaela La Regina, Stefano De Carli, Laura Rasero, Claudia Gatta, Giovanna Pentella, Gabriella Bordin, Antonella Croso, Annamaria Bagnasco, Gualberto Gussoni, Daiana Campani, Erica Busca, Danila Azzolina, Alberto Dal Molin, on behalf of the INTENTO Study Group

**Affiliations:** 1 Health Professions’ Direction “Degli Infermi” Hospital, 13875 Ponderano, Italy; claudia.gatta@aslbi.piemonte.it (C.G.); antonella.croso@aslbi.piemonte.it (A.C.); 2 Department of Health Sciences, School of Medical and Pharmaceutical Sciences, University of Genoa, 16132 Genoa, Italy; gianluca.catania@edu.unige.it (G.C.); annamaria.bagnasco@unige.it (A.B.); 3 Center for Health Outcomes and Policy Research, School of Nursing, University of Pennsylvania, Philadelphia, PA 19104, USA; 4 Research Department, FADOI Foundation, 20123 Milan, Italy; annachiara.crespi@gmail.com (A.C.); gualberto.gussoni@gmail.com (G.G.); 5 Internal Medicine Department, “Buon Consiglio” Fatebenefratelli Hospital, 80123 Naples, Italy; andreafontanella52@gmail.com; 6 Internal Medicine Department, “San Giovanni Calibita” Fatebenefratelli Hospital, 00186 Rome, Italy; dario.manfellotto@afar.it; 7 Clinical Governance and Risk Management, Healthcare Planning and Management Control, ASL 5 Liguria, 19124 La Spezia, Italy; micaela.laregina@asl5.liguria.it; 8 Internal Medicine Department, “San Antonio” Hospital, Azienda Sanitaria Universitaria, Friuli Centrale (ASU-FC), 33038 San Daniele del Friuli, Italy; decarli.stefano@gmail.com; 9 Department of Health Professions “Careggi” University Hospital, 50134 Florence, Italy; l.rasero@unifi.it; 10 Department of Health Sciences, University of Florence, 50139 Florence, Italy; 11 Health Professions’ Direction “Buon Consiglio” Fatebenefratelli Hospital, 80123 Naples, Italy; pentella.giovanna@fbfna.it; 12 General Medicine, “San Giacomo” Hospital, Castelfranco Veneto, 31033 Treviso, Italy; g.bordin@libero.it; 13 Department of Translational Medicine, University of Piemonte Orientale, 28100 Novara, Italy; daiana.campani@uniupo.it (D.C.); danila.azzolina@uniupo.it (D.A.); alberto.dalmolin@med.uniupo.it (A.D.M.); 14 “Maggiore della Carità” University Hospital, 28100 Novara, Italy; erica.busca@uniupo.it

**Keywords:** accidental falls, pressure ulcers, internal medicine, nursing care, randomised controlled trial, safety

## Abstract

The aim of the study was to explore the effects of Intentional Rounding, a regular-based proactive patient monitoring, on falls and pressure ulcers in internal medicine units. This is a cluster-randomised controlled study, where units were assigned (1:1) to Intentional Rounding (intervention group) or Standard of Care (control group). The primary outcome was the cumulative incidence of falls and new pressure ulcers. These events were considered separately as secondary endpoints, together with the number of bell calls and the evaluation of patient satisfaction. Primary analyses were carried out on the modified intention-to-treat population (hospitalisation of at least 10 days). Recruitment occurred between October 2019 and March 2020, at which time the study was prematurely closed due to the COVID-19 pandemic. Enrolment totalled 1822 patients at 26 sites; 779 patients were included in the modified intention-to-treat analysis. The intervention group had a lower risk of falls (adjusted incidence rate ratio 0.14; 95% confidence interval, 0.02–0.78; *p* = 0.03). There were no statistical differences in new pressure ulcers or the cumulative incidence of both adverse events. Mean bell calls for each patient were 15.4 ± 24.1 in the intervention group and 13.7 ± 20.5 in the control group (*p* = 0.38). Additionally, patient satisfaction in the intervention group was almost at the maximum level. Our study supports the usefulness of Intentional Rounding in a complex and vulnerable population such as that hospitalised in internal medicine units.

## 1. Introduction

In recent years, the healthcare setting has become very dynamic, and it requires healthcare professionals to explore new strategies to provide high-quality, evidence-based care that focuses on patient needs, patient satisfaction, and time-cost efficiency [1,2].

Patients hospitalised for acute diseases or exacerbation of chronic diseases often require intensive treatment and complex nursing interventions. Depending on the level of severity of the disease, the requests made to health professionals from patients can vary; often, the response to non-urgent requests [3], which may correspond to fundamental needs [4], are provided when there is time.

The 2013 Francis report highlighted how fundamentals of care have been disregarded in recent years, and how failures in this area are reflected in the dignity of inpatients and the quality of care provided, resulting in negative clinical outcomes (e.g., accidental falls and pressure injuries) [5,6]. In recent years, the issue of lack of fundamental care has become of particular importance and interest at the international level [7]. Although healthcare agencies such as the European Society for Quality in Healthcare in Europe and the Agency for Healthcare Research and Quality in the United States delivered recommendations to improve fundamental care for patients, poor practices still exist [8]. One of the recommendations of the Francis report for improving care and making up for missed care in acute settings is to systematise patient–nurse interactions through regular “rounds” [9]. This recommendation finds its application in Intentional Rounding (IR), a care approach developed in the USA by the Studer Group [5]. IR is regular-based proactive patient monitoring aimed at anticipating the satisfaction of fundamental needs and reducing negative nursing-sensitive patient outcomes, and increased interactions and frequent open communication are the key to engender a trust relationship between patients, families, and nurses and create a safer environment that can reduce anxiety from the hospitalisation and improve their satisfaction [3,10]. The concepts of “intentional” and “proactivity” make IR a well-structured process with specific objectives and differentiate IR from the “nursing rounds” historically performed in wards and considered devoid of a scientific basis [11].

This study was conducted within the fundamentals of care framework [9], where the context is an influencing factor to improve patient satisfaction. With the fundamentals of care, nurses should exteriorise the nursing contribution to patient care thorough developing a positive relationship between the nurse and patient. We took action on contextual factors to let leadership shape and influence processes of patient nursing care on the basis of dignity and respect.

Despite this, the literature reports that IR is not free of barriers and criticisms, and is related to an increased workload; moreover, some nurses report that it can limit the nurse’s decisional and critical capacity by making the work routine [2,3].

The proactive method IR has been the subject of interest for the past decade, particularly in the United States, as the country of origin of this method, and more generally in the English-speaking world [12]. Nevertheless, the results are fragmented, their generalisation difficult, and there is a lack of evidence of the applicability in other healthcare systems; furthermore, some of these studies lacked information on the methods for implementation of IR [13] (e.g., the frequency of rounds, if made by nurses only or not). These premises form the basis of the need for new robust research projects in this area, as indicated by the literature [10,13]. In this perspective, we carried out this study in order to analyse the effect of IR in clinical practice, focusing on an important setting, internal medicine wards, characterised by patients who are often complex [14] and require a care approach that includes not only clinical but also emotional management. 

## 2. Materials and Methods

### 2.1. Study Design

The FADOI-INTENTO is a cluster-randomised, controlled study (where the cluster was the hospital ward) promoted by the Italian Scientific Society of Internal Medicine, FADOI (Federazione delle Associazioni dei Dirigenti Ospedalieri Internisti), in collaboration with the National Association of Nurses of Medicine, ANìMO (Associazione Nazionale Infermieri Medicina Interna Ospedaliera).

### 2.2. Participants

The study sites were selected to be representative of internal medicine wards in Italy in terms of geographical distribution, hospital size, and level of care intensity. All patients aged 18 or above and consecutively admitted to the participating internal medicine wards were eligible for the study. No specific exclusion criteria were applied except refusal of consent. All enrolled patients were appropriately informed about the study procedures and aims and were requested to sign a written consent.

### 2.3. Randomisation and Masking

Twenty-eight hospital wards were selected by the scientific societies FADOI and ANIMO in order to be representative of the geographical distribution and level of care intensity of internal medicine units in Italy. These wards were randomly assigned (1:1 ratio) to the intervention or Intentional Rounding (IR) (n = 14) or Standard of Care (Soc) arm (n = 14) using a predefined list and the order of selection of the centres for the study.

Due to issues raised by local authorities (ethics committee and hospital administration), two sites allocated to the SoC arm were unable to participate. Patients were therefore enrolled in 14 and 12 sites in the IR and SoC group, respectively. Blinding was not applicable in this study.

Participants were recruited from October 2019 to March 2020.

### 2.4. Procedures

The IR study was delivered in 2 phases: preliminary and active phase. In the preliminary phase, the intervention sites received specific training to familiarise themselves with the study procedures and how to complete the IR log. The IR log collected data assessed along the IR (time of round, activities performed, and the signature of the nurse or nurse assistant) [15]. The training was carried out through a four-hour interactive meeting led by an expert in the IR nursing care model, with the participation of the nurses and nursing assistants (the minimum attendance had to be ≥65% of the staff). The IR was structured with rounds every 2 h throughout the 24 h of the day, performed alternately by either nurses or nursing assistants working at the site. IR is based on the assessment of the “4Ps” [5]: 1. positioning: make sure the patient is in a comfortable position and evaluate the risk of pressure ulcers; 2. personal needs: assess patient needs (e.g., to use the bathroom, to change incontinence pad, hydration); 3. pain: ask patients to describe their pain level on a scale of 0 to 10; 4. possession: make sure that any item a patient needs (phone, TV remote control, glass of water, or call bell) are within easy reach. During each IR, the healthcare professionals had to: introduce themselves to patients and their family who were in the room; carry out activities of the individual care plan and perform the “4Ps” assessment. Before leaving the room, the healthcare professionals had to ask the patients: “Is there anything else I can do for you?”; further, they would share information with patients about when the next round was scheduled, and report and sign the outcome of the round on the IR log. The IR performed during night shifts included only visual safety checks when patients were asleep. After completing the training session, the healthcare professionals piloted the IR procedures for a week. During this period, the local study supervisor performed sample checks of the IR log on alternate days, with subsequent feedback (positive or negative) to the healthcare professionals. During the study, the local study supervisor checked the correct completion of the rounds as recorded in the IR log.

### 2.5. Outcomes

The primary outcome of the study was the cumulative incidence of falls, defined as “an unexpected event in which the participants come to rest on the ground, floor or lower level” [16], and new pressure ulcers defined as “localized injury to the skin and/or underlying tissue usually over a bony prominence, as a result of pressure, or pressure in combination with shear”. Pressure ulcers were graded according to the NPUAP/EPUAP classification [17]. Secondary outcomes were the number of bell calls, the measurement of patient satisfaction with the nursing care received, and the nurses’ ability for critical thinking. Critical thinking was not possible to investigate due to impossibility to enrol nurses during the pandemic state.

### 2.6. Sample Size Estimation

The estimates for the calculation of the sample size were made on the basis of a pilot study performed at the coordinating centre. With a two-tailed test (Wald or maximum likelihood ratio) on the null hypothesis H0: incidence rate ratio (IRR) = 1 compared to the alternative hypothesis Ha: IRR ≠ 1, it was calculated that samples of 485 subjects in the IR group and 485 subjects in the SoC group allowed a power of 80% to detect an IRR of 0.5 (i.e., a 50% reduction), assuming an incidence rate in the IR group equal to 20 events per 1000 person-days, an average exposure time in both groups of 5 days, the dispersion parameter of the statistical model used (negative binomial model) being equal to 1.0 (conservative estimate), and the level of significance (alpha) being 0.05.

### 2.7. Data Analysis

Primary analyses were carried out on the modified intention-to-treat (mITT) population, which included the set of patients who had a hospitalisation of at least 10 days. Baseline descriptive statistics of patients according to the intervention and control group are reported. Continuous data are summarised as mean (standard deviation, SD) and categorical data are reported as absolute frequencies and percentages. Wilcoxon-type tests were performed for continuous variables and the Pearson chi-square test, or Fisher’s exact test, whichever was appropriate, was performed for categorical ones. The IRRs for the clinical trial endpoints were computed considering, for the person-time calculation, the in-hospital length of stay. The model-adjusted IRRs were also estimated together with the 95% confidence interval. A negative binomial linear mixed model (LMM) was computed considering an offset term on the logarithm of person-time (in-hospital stay) and a random intercept term accounting for the within-centre correlation. Computations were performed using R 3.6.2 language programming with lme4 packages.

### 2.8. Data Collection

In the active phase, in both the intervention and control sites, the following core information was collected for each patient at specified time-points: occurrence of falls and pressure ulcers and number of bell calls, twice a week and at discharge; patient satisfaction, only at discharge. Information on patient satisfaction was collected from patients who were able to answer two questions concerning the quality of nursing care and on the overall hospital stay. For each of these two questions, there was a score ranging from 1 (bad) to 7 (excellent). At the end of the project, the nurses involved in the project would be given a questionnaire for the evaluation of critical thinking. Other details on the characteristics of patients were collected at the time of admission to hospital including: age, sex, origin (home or other), presence of caregiver, chronic or acute active diseases, Barthel Index score [18], history of falls, and Morse Scale score [19]. The characteristics of patients were collected by means of an electronic case report form created ad hoc for the study, and the medical records were the source document. To collect the number of bell calls, a specific document called a call bells log was created [15].

## 3. Results

### 3.1. Patients

**Figure 1:**
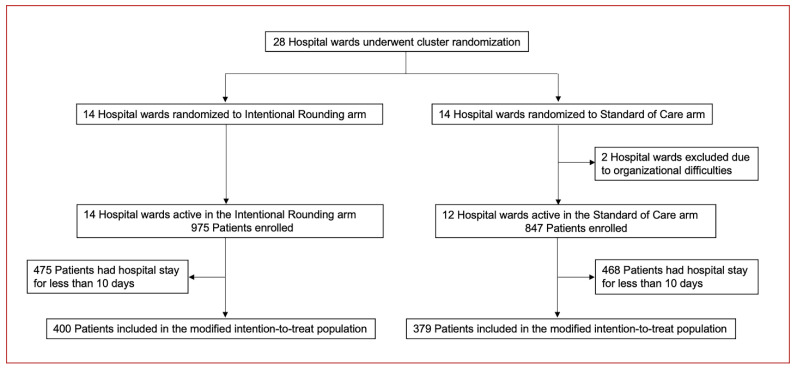
Trial profile. The modified intention-to-treat population included the set of patients who had a duration of hospitalisation of at least 10 days.

From October 2019 to March 2020, a total of 1822 patients were enrolled at 26 study sites; 779 patients were included in the mITT analysis (Figure 1).

Enrolment for the study ended concurrently with the development of the COVID-19 pandemic. This decision was made by the Steering Committee in consideration of the transformation of some wards participating in the study into units for patients with COVID-19; the continuation of the study would have resulted in the enrolment of patients with very different characteristics compared to those that had occurred during the study. The pandemic also prevented us from carrying out the nurse critical thinking evaluation since we believed that the concomitant and very complex professional condition would have affected the reliability of the results of the survey in an unacceptable way.

The demographic and clinical characteristics of the mITT population are reported in Table 1. Mean observation periods (SD) were 15.5 (6.3) days and 17.5 (10.2) days in the IR and SoC group, respectively (*p* = 0.08). Patients in the IR arm were significantly older (*p* < 0.0001), were more frequently affected with respiratory diseases (*p* = 0.001), had worse functional ability (Barthel Index) (*p* = 0.001), and had a higher risk of falls according to the Morse Scale (*p* < 0.0001) and history of falls (*p* = 0.01). On the other hand, patients in the SoC group were more frequently affected with endocrine or metabolic diseases (*p* = 0.002). The characteristics of patients observed in the overall study population are reported in the Supplementary Materials (Table S1).

### 3.2. Study Outcomes

The primary outcome of cumulative incidence of falls and new pressure ulcers accounted for 28 events in the IR group and 42 events in the SoC group (adjusted incidence rate ratio 0.50; 95% confidence interval (CI), 0.15 to 1.67; *p* = 0.26) (Table 2). Patients with at least one fall or new pressure ulcer were 20 out 400 patients (5%) in the IR group and 21 out of 379 patients (6%) in the SoC group (*p* = 0.73). In total, 4 and 19 episodes of falls occurred in patients in the groups IR and SoC, respectively (adjusted incidence rate ratio 0.14; 95% CI, 0.02 to 0.78; *p* = 0.03). The numbers of new pressure ulcers reported were 24 for IR and 23 for SoC (adjusted incidence rate ratio 1.00; 95% CI, 0.26 to 3.60; *p* = 0.98).

**Figure 2:**
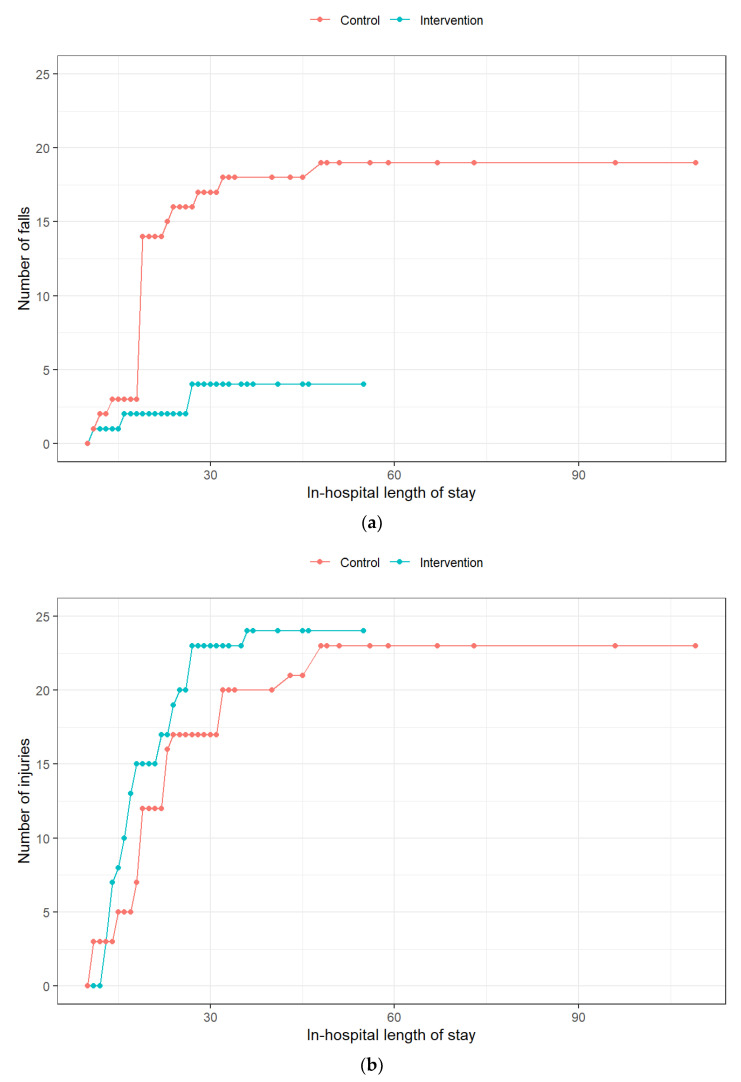

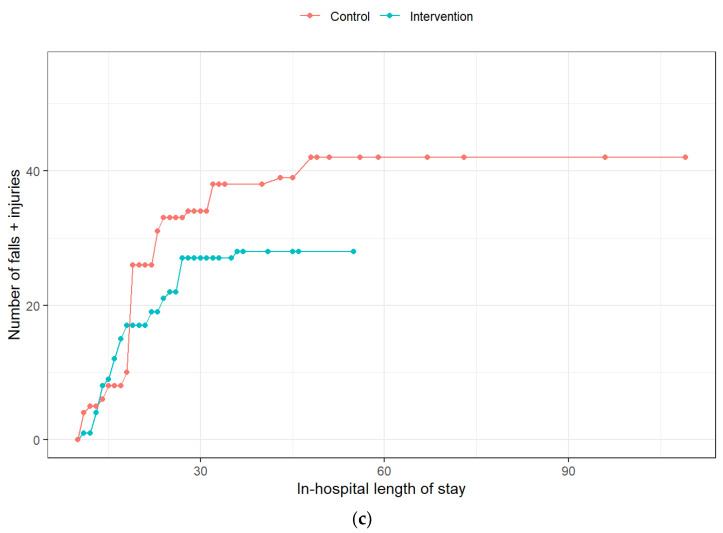
Cumulative number of hospital stay events in the two patient populations. (**a**)—falls; (**b**)—new pressure ulcers; (**c**)—composite endpoint.

Cumulative numbers of events (falls, new pressure ulcers, and their combination) during hospital stays are provided in Figure 2.

Data on occurrence of falls and pressure ulcers in the overall study population are reported in the Supplementary Materials (Table S2).

The mean number (SD) of bell calls for each patient was 15.4 ± 24.1 in the IR group and 13.7 ± 20.5 in the SoC group (*p* = 0.38). The patient satisfaction questionnaire presented an average score of 13.25 ± 1.72 for IR and 12.17 ± 1.87 for SoC (*p* = 0.87).

## 4. Discussion

The search for care approaches that may increasingly meet patients’ needs, and that can be manageable for healthcare professionals, is a challenging issue. Our study was designed to evaluate whether the application of a proactive approach could effectively meet safety and fundamentals of care, which constitute the cornerstones of the nursing profession.

We chose to use the randomised controlled trial approach because, as indicated by the literature, this is “the best design to achieve an unbiased estimate” of the impact of a complex experimental intervention [20].

Furthermore, to evaluate the effectiveness of interventions, a composite outcome including the occurrence of falls and new pressure ulcers was considered, in an original way compared to the majority of previous studies on the subject. We adopted this strategy based on the concept that a composite outcome can offer a more comprehensive evaluation of the effectiveness of the experimental intervention [21]. This is also based on the fact that the events with which we chose to compose the main outcome are of primary interest for patients, in addition to nursing-sensitive patient outcomes [22] and indexes of good quality and safety of care. Nursing-sensitive indicators play a key role in assessing the nursing care and the performance of a nursing organisational intervention [23,24].

However, to better understand the specific impact of the experimental intervention on each component of the composite outcome, as already done in other trials that used this methodological approach [25], we further analysed each component individually, considering them as secondary endpoints.

In our cluster-randomised, controlled study, the proactive method IR reduced by about 50% the incidence of a composite endpoint including falls or new pressure ulcers during hospital stay versus SoC (28 vs. 42 events, respectively); the effect was mostly related to a relevant and statistically significant difference of incidence of falls (4 and 19 events in the IR and SoC arm, respectively).

Patient safety is a major objective for the implementation of a nursing care pattern, and the occurrence of falls has been investigated in a number of studies assessing the effectiveness of IR [4,26,27,28,29]. Significant reductions in the incidence rate of falls of around 50% have been reported in a few studies [26,27,29], whilst not statistically significant but clinically relevant reductions have been observed in other trials [4,29]. Our results are comparable with these previous findings, to an even higher extent, with the observed relative risk reduction in the IR group being around 80%. Moreover, this result was achieved despite the fact that the IR study population appeared to be at a higher risk of falling if compared with controls, according to general characteristics (e.g., age), the Morse Scale, and the history of previous falls. Such a relevant effect on falls confirmed that IR may have a positive impact on patient safety as well as a positive economic impact. Indeed, a lower rate of falls reduces the risk of longer hospital stays due to this complication, as well as the need for additional exams and treatments and possible requests for damage compensation [27].

Pressure ulcers are one of the most reported nursing-sensitive patient outcomes, with rates ranging from 5% to 32% of patients, and an estimated mean prevalence of 21% in acute settings [30]. Through systematic control and stimuli to mobilisation (both active and passive), IR can play a significant role in the prevention of these complications. However, few studies have measured this outcome [15,26], and the available results are heterogeneous. In our experience, the overall rate of occurrence of new pressure ulcers was lower if compared with previous reports, and no difference was seen between IR and SoC. In interpreting this finding, it must be considered that the attention towards the prevention of pressure ulcers is generally high also in the SoC, with the adoption of measures such as patient mobilisation and the use of anti-decubitus mattresses. Moreover, we cannot rule out that the awareness of participating in a clinical study further increased the attention towards this outcome, particularly in the SoC group. The resulting low level of occurrence of pressure ulcers in the SoC group reduced the possibility of identifying a potential positive effect of IR. Further, and as a limitation of the study, it is probably not trivial to consider that the patients in the intervention group had a higher rate of falls and lower functional ability than patients in the control group.

Although less clinically relevant than new pressure ulcers or falls, the phenomenon of calling the staff is one of the most investigated outcomes related to IR. The potential opportunity offered by IR is to anticipate the needs of the person and therefore achieve a greater satisfaction with the assistance received [13]; this can also lead to less necessity to call for nurses. As summarised in the review by Ryan et al. [10], four out of six studies which evaluated these items registered a statistically significant reduced number of bell calls associated with IR. On the other hand, less clear evidence has been reported for more rapid responses by the nurses [31]. Unlike most previous studies, in our experience, the number of bell calls was similar and even slightly higher in the IR group compared with SoC. A possible explanation for this result can be based on the type of patients assisted. Indeed, the literature indicates that this outcome is very sensitive to cognitive state [3], which is frequently impaired among patients hospitalised in internal medicine wards. Further, the different in health status of patients randomised to IR in our study may have generated a greater need for nursing care, and therefore of bell calls. Lastly, previous studies have reported frequent incorrect execution of the rounds, specifically with the final sentence “Is there anything else I can do for you? I’ll be back in …” [32]. If the healthcare professional does not return after the promised time, the patient may lose confidence in the healthcare professional and in the experimental intervention, thus triggering a sense of anxiety which leads to more frequent calls for assistance [31]. The preliminary phase of training for healthcare professionals that we performed was aimed at optimising the execution of the rounds and therefore minimising this possibility, but we cannot rule out that this failure also occurred in our study.

The perceived satisfaction with the nursing care received is an additional point to consider while implementing a new method aimed at improving the quality of care. In the specific case of IR, this pattern of care could lead to an increase in satisfaction since patients perceive that the nurse is really interested in their care needs, both physical and emotional, thus feeling more peaceful and secure [33,34]. On the other hand, it was reported that some patients can consider the proactive “control” of IR as intrusive and disturbing [3]. The results of previous studies have documented that IR is associated with a significant increase in patient satisfaction [4,10,26,29]. A higher level of patient satisfaction with IR vs. SoC was also observed in our study, although the difference was not statistically significant. However, note that the level of satisfaction was already very high even for patients in the SoC group and close to maximum, and therefore the improvement margin for IR was in fact limited. This finding is a positive aspect for nursing care in IMUs in Italy, but we cannot exclude the possibility that the awareness of participating in a clinical study may have contributed to it at least in part. Indeed, study participation can have a positive impact on the clinical practice [35].

The introduction of IR has brought with it doubts and perplexities that have been discussed in the scientific literature [2,3], such as the possibility that it increases the workload too much, or that not all patients have to undergo IR. Our study was not designed to address these issues, and therefore we are not able to provide insights on these matters. Another issue raised by some authors is that IR reduces the critical thinking and decision-making ability of the nurse with respect to the assistance to be provided to the person [2,3]. We had planned to investigate this aspect in our study through a specific questionnaire, but unfortunately, the onset of COVID-19 prevented us from gathering reliable feedback, in consideration of the critical professional and psychological conditions of the nursing staff during the pandemic. However, we consider this aspect of particular importance, and we believe it should be addressed in future studies concerning IR.

Our results suggest that IR can be implemented in clinical practice. Based on our experience, to overcome barriers and for the successful implementation of IR, the leadership and the education play a key role [3,10,36]; in fact, as indicated by the literature [37], to implement a complex intervention, such as IR, specific training is crucial. Pre-registration nursing students, if involved in IR and through good education, can benefit through professional growth by increasing their communication skills, needs assessment, and in assuming responsibility towards the patient in their future clinical practice [36].

### Limitations

The study has some points worth discussing and that can be considered possible limitations. First is the choice of the composite outcome. The literature indicates three recommendations for the ideal use of composite endpoints: the outcomes must be of similar importance for the patient, be of similar frequency, and have the same degree of sensitivity to the intervention [25]. In our study, pressure ulcers had a much higher frequency and a much lower sensitivity to IR than those observed with falls, and this may have created a bias in the result of the outcome. However, the highly significant result obtained for the endpoint of falls appears, in our opinion, to be of clinical importance and the results of our study to be of potential interest of for the scientific community. Secondly, to use the mITT population for assessment of the primary outcome might have exposed the work to attrition bias, but it was thought that to detect possible adverse events, it was important to include only subjects exposed to the experimental intervention or SoC for a sufficient period of time. In any case, no significant differences were observed for possible confounders by comparing the baseline characteristics of patients in the mITT and overall study populations. Further, our choice probably did not substantially influence the statistical power of the study since the statistical assessment of clinical endpoints was qualitatively consistent between the mITT and overall study populations and did not prevent the possibility of detecting a statistically significant difference for falls [38]. The mITT analyses are consistent with ITT approach (Table S1). Third, the two study groups differed in some baseline characteristics. Unfortunately, this is a risk that can occur with the application of the cluster randomisation. In our study, the application of an individual randomisation would have led to significant organisational problems, risk of errors, and possible discomfort in relations with patients (and caregivers) residing in the same room but treated with different care methods. The presence of different characteristics between the study populations can certainly complicate the interpretation of the results obtained. However, in our study, patients randomised to IR had a higher risk profile for complications, and this supports the results obtained with IR and the validity of the intervention. Fourth, the impossibility of blinding may have introduced the risk of detection bias and performance bias. Our efforts were directed to minimise these risks by means of strict protocol recommendations and specific training. Furthermore, falls, which represented the most sensitive outcome to IR, are not influenced by the aforementioned detection bias. Fifth, the onset of the COVID-19 pandemic prevented the study from achieving the expected sample size. We cannot exclude the possibility that this slightly influenced the outcome of the research, but it is likely that the results obtained on the various endpoints would not have been qualitatively different.

## 5. Conclusions

In conclusion, our study provides new data to support the usefulness of IR in addressing fundamentals of care, in particular in a complex and vulnerable population such as that hospitalised in internal medicine wards. The significant effect of reducing falls among patients assisted with IR represents an important clinical element in favour of patient safety and the adoption of this method. Future studies should further strengthen the evidence in this direction and address the open issues related to the implementation of IR, such as the profile of the patient who can benefit most from this method, as well as the workload associated with IR and its impact on the recovery of physical activities, self-government, and the critical thinking and decision-making ability of nurses.

## Figures and Tables

**Table 1:** Baseline characteristics of the modified intention-to-treat population.

	Intentional Rounding (n = 400)	Standard of Care (n = 379)	*p*-Value
Age (years)	81 (74–87)	77 (69–84)	<0.0001
Sex			0.994
Male	187 (47%)	178 (47%)	
Female	213 (53%)	201 (53%)	
Origin			0.147
Home	311 (78%)	316 (83%)	
Other	89 (22%)	63 (17%)	
Chronic/acute diseases			
Cardiovascular	228 (57%)	216 (57%)	0.966
Endocrine/metabolic	116 (29%)	149 (39%)	0.002
Gastroenteropancreatic	62 (16%)	64 (17%)	0.587
Malignancy	84 (21%)	91 (23%)	0.304
Neuropsychiatric	71 (18%)	50 (13%)	0.082
Osteoarticular	80 (20%)	71 (18%)	0.669
Respiratory	153 (38%)	104 (27%)	0.001
Barthel Index	45 (15–75)	55 (25–95)	0.001
History of falls	106 (27%)	72 (19%)	0.011
Morse Scale	35 (25–55)	35 (15–50)	<0.0001
Length of hospital stay (days)	14 (12–17)	14 (11–19)	0.080
10–15 days	266 (66%)	222 (59%)	
16–20 days	74 (19%)	79 (20%)	
More than 20 days	60 (15%)	78 (20%)	

Continuous data are reported as median (interquartile range) and categorical data as absolute frequencies (percentage). Wilcoxon-type tests were performed for continuous variables and the Pearson chi-square test, or Fisher’s exact test, whichever was appropriate, was performed for categorical variables.

**Table 2:** Study outcomes in the modified intention-to-treat population.

Outcomes	IR (N° of Events)	CG (N° of Events)	Observed IRR	NBM Adjusted IRR (95% CI)	*p*-Value
Composite outcome (Falls + bedsores)	28	42	0.7	0.5 (0.15–1.67)	0.26
Falls	4	19	0.22	0.14 (0.02–0.78)	0.03
Bedsores	24	23	1.1	1 (0.26–3.60)	0.98

Legend: CG—control group; IR—Intentional Rounding; IRR—incidence rate ratio; NBM—negative binomial model. The person-time in the modified intention-to-treat population was 6207 and 6537 days in the Intentional Rounding and Standard of Care group, respectively.

## Data Availability

Not applicable.

## Supplementary Materials

The following supporting information can be downloaded at https://www.mdpi.com/article/10.3390/jcm11143976/s1. All data relevant to the study are included in the article or uploaded as supplementary materials.

## Appendix A

Members of the INTENTO Study Group:

OSPEDALE DI CEVA-ASL CN1-CUNEO

- Mara Luzzo (investigator);
- Paolo Alicandri;
- Alberto De Paoli;
- Carlo Lorenzo Muzzulini.

AZIENDA OSPEDALIERO UNIVERSITARIA CAREGGI-FIRENZE

- Anna Maria Tognoni (investigator);
- Luciana Zetti;
- Anna Maria Esposito;
- Anna Brachi;
- Alessandra Lombardi;
- Annalisa Pelosi.

OSPEDALE SAN GIOVANNI CALIBITA FATEBENEFRATELLI-ROMA

- Simone Gatti (investigator);
- Marco Frisardi;
- Dario Manfellotto;
- Federica Deci.

OSPEDALE CIVILE SS ANNUNZIATA-ASL CN1-SAVIGLIANO

- Federica Fissore (investigator);
- Lara Fiorenza Gosmaro;
- Bruna Bassetti;
- Giovanni Gulli;
- Fabiola Dutto;
- Alice Tidona.

OSPEDALE DI RHO-MILANO

- Laura Zoppini (investigator);
- Loredana Viviano;
- Maria Teti;
- Alessandro Vismara;
- Cristina Sanguini;
- Stefano Palumbo.

AZIENDA OSPEDALIERO UNIVERSITARIA CAREGGI-FIRENZE

- Stefano Catalano (investigator);
- Moreno Ceccherini;
- Provvidenza Solamino;
- Sabrina Fieri;
- Talisa Bacci.

OSPEDALE DI CASTELFRANCO VENETO-ULSS 2 MARCA TREVIGIANA

- Gabriella Bordin (investigator);
- Federica Zamperin;
- Sabina Villalta;
- Nicoletta Conte;
- Elisabetta Cavallin.

OSPEDALE DI RHO-MILANO

- Laura Zoppini (investigator);
- Loredana Viviano;
- Stefano Beretta;
- Alessandro Vismara;
- Elena Landriani;
- Elisabetta Galbier.

AZIENDA OSPEDALIERA “SS. ANTONIO E BIAGIO E CESARE ARRIGO” ALESSANDRIA

- Tatiana Bolgeo (investigator);
- Lorella Gambarini;
- Loredana Grantini;
- Aldo Bellora;
- Antonio Maconi.

OSPEDALE CIVILE SS ANNUNZIATA-ASL CN1-SAVIGLIANO (CN)

- Federica Fissore (investigator);
- Alessandro Parizia;
- Bruna Bassetti;
- Giovanni Gulli.

OSPEDALE CIVILE RAMAZZINI-CARPI (MO)

- Barbara Ferrari (investigator);
- Lorella Rossetti;
- Carlo Di Donato.

OSPEDALE DI NOVENTA VICENTINA-VICENZA

- Adriana Dal Maso (investigator);
- Paola Lovo;
- Paola Birro;
- Michela Muriago.

BUON CONSIGLIO FATEBENEFRATELLI-NAPOLI

- Maria Fiscale (investigator);
- Elena Grimaldi;
- Andrea Fontanella.

OSPEDALE DI MONTEBELLUNA-ULSS 2 MARCA TREVIGIANA-TREVISO

- Katia Gazzola (investigator);
- Silvia Feltrin;
- Ernesto De Menis.

OSPEDALE SAN BORTOLO-ULSS 8 BERICA-VICENZA

- Renato Moresco (investigator);
- Alessandra Rampazzo;
- Francesca Baldi;
- Giovanni Scanelli.

OSPEDALE DEGLI INFERMI DI PONDERANO-ASL BIELLA-BIELLA

- Dino Stefano Di Massimo (investigator);
- Irene Corniati;
- Fabio Bertoncini;
- Aldo Tua.

AZIENDA OSPEDALIERO UNIVERSITARIA SENESE-SIENA

- Teresa Troisi (investigator);
- Fabrizio Di Pace;
- Pier Leopoldo Capecchi.

CASA DI CURA BEATO PALAZZOLO-FONDAZIONE DON GNOCCHI MILANO

- Monica Talpos (investigator);
- Maria Concetta La Corte;
- Elisabetta Frontini;
- Pierluigi Gnocchi.

OSPEDALE SAN DONATO-USL SUD EST AREZZO

- Stefania Francioni (investigator);
- Alice Zacchei;
- Annunziata Zuccone;
- Mario Felici.

OSPEDALE SAN PAOLO-ASL 2 SAVONESE-SAVONA

- Roberta Rapetti (investigator);
- Elena Sartori;
- Barbara Zanella;
- Lionello Parodi.

PRESIDIO OSPEDALIERO SANTA CROCE-AZIENDA OSPEDALI RIUNITI MARCHE NORD-FANO (PU)

- Serena Frassini (investigator);
- Donatella Giovannini;
- Sabrina Bartoloni;
- Gabriele Frausini.

POLICLINICO SAN MARTINO-GENOVA

- Gianluca Catania (investigator);
- Elvira Tedesco;
- Giulia Alessandri;
- Giovanni Passalacqua.

OSPEDALE BUCCHERI LA FERLA FATEBEBEFRATELLI-PALERMO

- Monica Cuccia (investigator);
- Gianfranco Lofaso;
- Fabio Cartabellotta.

OSPEDALE S. ANDREA MASSA MARITTIMA (GH)

- Roberta Petri (investigator);
- Barbara Proja;
- Massimo Alessandri.

AZIENDA OSPEDALIERA OSPEDALI RIUNITI VILLA SOFIA-CERVELLO (PA)

- Maria Giovanna Curatolo (investigator);
- Ignazia Lo Burgio;
- Gabriele Nicolosi;

PRESIDIO OSPEDALIERO VALDESA CAMPOSTEGGIA-SIENA

- Stefano Trapassi (investigator);
- Federica Cavallaccio;
- Maria Claudia Cerza;
- Emanuela Corsi;
- Valerio Verdiani.

STUDY SUPPORT

- Irene Zanchetta (ASL BIELLA);
- Valentina Prior (ASL BIELLA);
Francesca Bider (MSN STUDENT-UNIVERSITA’ DEL PIEMONTE ORIENTALE).

Steering Committee members: Dino Stefano Di Massimo, Alberto Dal Molin, Gianluca Catania, Andrea Fontanella, Micaela La Regina, Stefano De Carli, Laura Rasero, Claudia Gatta, Giovanna Pentella, Gabriella Bordin, and Antonella Croso.
