# Supplementary material for: Intentional Rounding versus Standard of Care for Patients Hospitalised in Internal Medicine Wards: Results from a Cluster-Randomised Nation-Based Study
Source: J Clin Med. 2022 Jul 8;11(14):3976. doi: 10.3390/jcm11143976 (PMC9320400; doi:10.3390/jcm11143976)
Supplement: Supplementary file 1 [file jcm-11-03976-s001.zip › jcm-1732181-supplementary.pdf]

SUPPLEMENTARY MATERIALS

Table S1: Baseline characteristics of the overall study population

	**Intentional Rounding (n=975)**	**Standard of Care (n=847)**
Age (years)	80 (71-86)	76 (66-83)
Sex		
Male	506 (52%)	431 (51%)
Female	469 (48%)	416 (49%)
Origin		
Home	830 (85%)	726 (86%)
Other	145 (15%)	121 (14%)
Chronic / acute diseases		
Cardiovascular	521 (53%)	440 (52%)
Endocrine / metabolic	263 (27%)	267 (32%)
Gastroenteropancreatic	152 (16%)	148 (17%)
Malignancy	184 (19%)	262 (31%)
Neuropsychiatric	166 (17%)	103 (12%)
Osteoarticular	159 (16%)	142 (17%)
Respiratory	331 (34%)	215 (25%)
Barthel Index	55 (20-95)	75 (35-100)
History of falls	225 (23%)	148 (17%)
Morse Scale	35 (20-50)	25 (15-45)
Lenght of hospital stay (days)	8 (5-13)	9 (6-14)

Continuous data are reported as median (interquartile range) and categorical data as absolute frequencies (percentage). Wilcoxon-type tests were performed for continuous variables and the Pearson chi-square test, or Fisher-exact test whatever appropriate, for categorical variables.

Table S2: Study outcomes in the overall study population

Outcomes	Intentional Rounding (N° of events)	Standard of Care (N° of events)	Observed IRR	Negative Binomial Model Adjusted IRR (95% CI)	P-value
Composite outcome (Falls + pressure ulcers)	42	47	0.86	0.7 (0.22-2.10)	0.52
Falls	8	22	0.35	0.25 (0.06-0.93)	0.04
Pressure ulcers	34	25	1.1	1.31 (0.35-4.87)	0.68

The Person Time in the overall study population is 9499 and 9152 days in the Intentional Rounding and Standard of Care group, respectively
